# Surgical Treatment of Dysmenorrhœa

**Published:** 1866-10

**Authors:** J. F. Miner


### ART. III—*Surgical Treatment of Dysmenorrhœa.*

BY J. F. MINER, M. D.

Painful menstruation, menorrhagia and amenorrhœa, the three principal deviations from the normal condition of this secretion, have been regarded with great uniformity of opinion as arising from functional disturbance, and not until quite recently, as in any way dependent upon organic or local change. More recently it is being believed that one of these conditions, dysmenorrhœa, is generally due to closure, obstruction or narrowing of the cervical canal. Dr. Sims in his new work upon Uterine Surgery says, "Menstruation may be attended by a general malaise, but should not, as a rule, be accompanied by any very severe degree of suffering. If there is much pain, either preceding its irruption or during the flow, there will generally be a physical condition to account for it, and this will be of a nature to obstruct mechanically the egress of the fluid from the cavity of the womb. The obstruction may be the result of inflammation and attending turgessence of the cervical mucous membrane, whereby this canal becomes narrowed merely by the tumefaction of its lining coat. But by far the most frequent cause of obstruction is purely anatomical and mechanical."

Monographs and journal reports have inculcated these views, and suggested surgical operation for relief, which has been adopted by many operators in uterine diseases, until it will not be uninteresting to consider somewhat the grounds upon which these opinions are based, and the results of the surgical interference recommended.

If, as is alleged, painful menstruation depends upon obstruction to the egress of the secretion from the cavity of the womb, why is it so common in women who have had the uterine neck dilated by the passage of a child at full time, and why is not *pain* the chief and important symptom, in cases of complete occlusion of the canal and retention of the menstrual secretion in the cavity of the womb? Can it be believed when the common uterine sound or No. 12 bougie is readily passed through the cervical canal, that the menstrual fluid meets with obstruction sufficient to produce what is termed dysmenorrhœa? Dr. Sims figures the uterus with anteflexion and narrowing of the canal; the cause of anteflexion and obstruction being a fibroid tumor "as large as an almond in the anterior wall," but in his manner of diagnosing fibroid tumors, he passes sounds, bougies, etc., etc., even when these growths are very large and fill not only the cavity of the womb but of the lower portion of the abdomen, the flexible instruments passing readily between the tumor and the thinned walls of the opposite side. It would appear probable that a fibroid tumor in the walls of the uterus, not larger than a filbert, would not obstruct the flow of thin fluid, either by its own pressure or by any flexion it would be likely to produce, but no one will believe that our present means of exploration enables us to determine the presence or absence of such a growth during life. In evidence that dysmenorrhœa is caused by obstruction, it is said that after dilatation, or incision of the neck, the pain generally subsides—that treatment proves the nature of the disease. In irritable conditions of the mucous lining of the urethra, every surgeon is familiar with the influence of allowing a carefully introduced cathater or bougie to remain for a short time each day, has observed relief from the pain of urination, which had been perhaps as severe, as painful menstruation. The relief obtained by incision, quite possibly, does not depend upon the enlargement of the outlet, but from local depletion or other easily understood influences.

It is not safe to settle this question, which has been so long left undetermined, upon insufficient ground, not so much on account of any differences of opinion which have prevailed, and do still prevail, as on account of the practical inferences which follow. If we adopt the view that pain at the menstrual molomen is due to obstruction in the uterine outlet, we at once set ourselves to devising means for its removal. If it appears that the cervical canal is too small, the questions of incision and dilatation at once present themselves. If flexions are looked upon as such causes, pessaries with uterine stems are brought in requisition, and so it comes at last, that we attack pain at time of menstruation, with cutting instruments, sponge tents, sea tangle, stem pessaries and the whole armament of uterine surgery.

In conditions of the most perfect health the period of ovulation is attended by more or less pain; those least effected experience a feeling of lassitude, fullness and weight in the pelvis; these symptoms are slight in some instances, and in others so severe as to constitute the disease in question. At first there is an unusual discharge of mucus, which soon turns rust colored from admixture of blood, and when this discharge is fully established, upon the second or third day, the pain abates. As the menstrual period comes on, a congestion takes place in the whole generative apparatus; in the fallopian tubes and the uterus, as well as in the ovaries themselves and their contents. One of the graafian follicles is more especially the seat of an unusual vascular excitement, and becomes distended by the fluid which accumulates in its cavity, and finally ruptures. This is as near description of the condition as can be gathered from recent physiologists, and is sufficiently minute for our present purpose. If the above is correct, it would seem to leave little ground for doubt as to the natural causes of pain, and would narrow down the "anatomical and mechanical" obstruction theory, to very infrequent and unusual cases. Painful menstruation is a very common disease, much more common than anatomical change in the os or cervix uteri. The idea that nearly all cases of this nature depend upon obstruction to the egress of the fluid secreted, is only equalled in extravagance by the remedy recommended—*incision of the neck.* While it may be proper in a few rare instances to make this operation, which is probably not very dangerous, yet to claim it as the only efficient and proper remedy for dysmenorrhœa, is a proposition to be investigated before being adopted.

The subjects of this disease are more commonly young females, in which the disease often disappears spontaneously in after years, when the menstrual secretion is fully established. That uterine tumors may be the cause of pain at the monthly period of congestion, is very natural, since no morbid growths could be forming in the womb without causing more or less irritation; but these growths are not often, the cause of pain at the monthly period in young females; they rarely appear until later in life. Fibroid tumors are not very frequent; if present they cannot be detected very early, if at any time, and flexions from such cause cannot be separated from flexions from other causes. It is easy to show by diagrams how a small fibroid tumor, not larger than a filbert, would cause retro or anteflexion, according to its location in the anterior or posterior wall of the womb, but it is more like the paper cities of a newly discovered country, than the actual conditions known to exist. If the presence of such growth, however, has been determined, we are not to conclude that the flexion which its situation outside the normal centre of gravity is supposed to produce, is the cause of pain either during menstruation or between the periods.

Leaving for a time menstrual obstruction, it would be interesting to consider contraction of the uterine neck as a cause of sterility, and incision as the sovereign remedy. These subjects hang together by a sort of affinity of treatment which makes it pertinent to group them, so that what may be said of incision for obstructed menstrual discharge, may also be said of incision of the uterine neck for all purposes in general. The influence of great names has brought this operation rapidly into practice, and it is adopted upon authority, rather than upon experience in its results. It is, however, necessary to test its efficacy, and with this view, its adoption to some extent, may be justifiable. This surgery has been made easy, by the invention of various instruments, the most fashionable and approved being constructed so that they may be first introduced into the womb, then the cutting blade protruded from its sheath, dividing the tissue from within outwards. Dr. Sims differs in this from the author of this practice, Dr. Simpson, and fixes the womb, making his incisions from without inwards. The very fact that these instruments can be introduced is sufficient evidence that fluid can escape, and is also sufficient proof that impregnation can take place, so far as the size of this opening may influence it.

What is the effect of incising the neck of the womb upon either side of the cervical canal? When the incision has healed, is the opening any greater? is the tissue any more yielding? is egress or ingress any more easy? What effect is produced upon other canals of somewhat similar nature, by incising their walls, and what does experience show to be the effect of incising the uterine neck? These questions are settled already upon the authority of the best men in this department of practice, but unfortunately for the profession and fortunately for the public, they are decided in different ways, though it must be confessed that a wonderful harmony prevails in the opinion that the canal must be in some way enlarged, the diversity of opinion being upon the manner it shall be done. That there are rare instances in which it might be proper to make incision of the uterine neck, will not be denied, but that this operation is required, when sounds, bougies, uterotomes, etc., etc., are easily introduced, is denied; and it is hoped physicians will judge of this for themselves, independent of the opinions of others.

That the operation can be made with safety there can be no doubt, but will it do any good, and is it not likely to do harm when it is made? The well known tendency in cicatrization of wounds to contraction is manifest in these cases more than in most others, and whoever examines the neck of the womb three months after incision, will find induration and contraction greater than before such operation. There is no known method of preventing the union of the incision and the subsequent contraction, and those who practice it, confess inability to prevent this result. Granting then that mechanical obstruction is the real cause of pain, or narrowing of the canal the barrier to conception, is incision to be depended upon as a sovereign remedy? Whoever believes it must test its efficacy, and their victims bear the experiment. I have seen patients a few months after this operation had been performed, never a whit better, always disgusted with what had been done for them, and more earnestly than ever seeking other means of relief. Experience is the sole basis of remark upon the efficacy and propriety of incision of the uterine neck for either of the purposes mentioned; if future observation should change opinion in this respect, and show the propriety of this practice—should demonstrate that in any considerable proportion of cases favorable results follow, nothing can afford greater pleasure than to accord to this operation the credit rightfully belonging to it. It has been proposed and is advocated by the most distinguished observers, and it seems almost like sacrilege to question the infalibility of such men. If physicians were at liberty to yield their opinions upon such questions to the dicta of others, without being first convinced of the correctness of their views, it would appear better to accept the teaching of great men, and be governed by distinguished names; but since truth is to be the only standard, so evidence and proof is to be the only governing influence. To repeat briefly: objection to this practice is based upon the idea that mechanical obstruction to the egress of the menstrual fluid is not the cause, in most instances of dysmenorrhœa, the natural and philosophical causes being indicated. Incision of the uterine neck for sterility is also opposed in all cases where bougies, sounds and uterotomes are easily passed, because if such instruments can pass, impregnation can take place so far as the size of the uterine canal is concerned. Granting the premises; incision is objected to, because the wound will rapidly cicatrize and contract and obstruction be increased rather than removed. Dilatation for similar purposes, when indicated, is less objectionable and may always be accomplished; though liable to contract like incision, it is still less likely to produce injurious effects; the canal, however, will almost always return in a short time, to its former, or nearly its former conditions.

